# Preparation of Bi_2_O_3_/Al Core-Shell Energetic Composite by Two-Step Ball Milling Method and Its Application in Solid Propellant

**DOI:** 10.3390/ma12111879

**Published:** 2019-06-11

**Authors:** Min Xia, Qifa Yao, Huilian Yang, Tao Guo, Xiuxin Du, Yanjie Zhang, Guoping Li, Yunjun Luo

**Affiliations:** School of Materials Science & Engineering, Beijing Institute of Technology, Beijing 100081, China; xminbit@bit.edu.cn (M.X.); yao18811316232@163.com (Q.Y.); luckylianer@foxmail.com (H.Y.); guo15600922209@163.com (T.G.); 13188782016@163.com (X.D.); 18511220289@163.com (Y.Z.)

**Keywords:** ball milling, Bi_2_O_3_/Al, propellant, density, density specific impulse

## Abstract

In this article, Bi_2_O_3_/Al high-density energetic composites with a core-shell structure were prepared by a two-step ball milling method using a common planetary ball milling instrument, and their morphology, structure, and properties were characterized in detail. Through a reasonable ratio design and optimization of the ball milling conditions, the density of the Bi_2_O_3_/Al core-shell energetic composite is increased by about 11.3% compared to that of the physical mixed sample under the same conditions. The DSC (Differential Scanning Calorimetry) test also showed that the exothermic quantity of the thermite reaction of the energetic composite reached 2112.21 J/g, which is very close to the theoretical exothermic quantity. The effect of Bi_2_O_3_/Al core-shell energetic composite on the energy performance of insensitive HTPE propellant was further studied. The theoretical calculation results showed that replacing the partial Al with Bi_2_O_3_/Al core-shell energetic composite can make the density of propellant reach 2.056 g/cm^3^, and the density specific impulse reach 502.3 s·g/cm^3^, which is significantly higher than the density and density specific impulse of the conventional composite solid propellant. The thermal test showed that the explosive heat of the HTPE (Hydroxyl terminated polyether) propellant also increased with the increase of the content of Bi_2_O_3_/Al core-shell energetic composite.

## 1. Introduction

The development of modern weapons and space transportation has placed increasing demands on the energy performance of solid propellants. High energy performance is the eternal target of the development of solid propellants technology [1]. The density specific impulse is an important performance parameter that directly reflects the energy performance of the propellants. For fixed volume loading requirements, the greater the density specific impulse is, the higher the total impulse of the propellants will be. Therefore, the application of high-density energetic components in solid propellants can significantly increase the thrust of solid rocket engines [2].

Aluminum (Al) is the most commonly used metal burning agent in high-energy solid propellants, which can increase the density and combustion heat of the propellants. Bismuth oxide (Bi_2_O_3_) is a kind of typical heavy metal oxide which can react with Al violently and produce heat by redox reaction, called the thermite reaction [3,4]. Thus, if Bi_2_O_3_ can be used as the oxidant component in the solid propellant, it is expected to impart high density specific impulse characteristics to the solid propellant. However, Bi_2_O_3_ is energy-free. How to give full play to the efficiency of the thermite reaction becomes the key technology of a high energy density propellant containing Bi_2_O_3_ [5,6]. In addition, when the polyurethane system is used as a solid propellant binder, the bismuth element can catalyze the reaction between isocyanate groups and hydroxyl groups, resulting in a sharp increase in the viscosity of the propellant slurry, which cannot be poured [7]. Therefore, the preparation of Bi_2_O_3_/Al core-shell energetic composites to effectively coat Bi_2_O_3_ with Al is a possible way to solve the above problems.

High-energy ball milling, also known as mechanochemistry, has become an important method to prepare ultra-fine materials. As a new technology, it can significantly reduce the activation energy of the reaction, refine the grains, and increase the powder activity and the uniformity of particle distribution. In addition, the technology can promote solid state ion diffusion, induce a low temperature chemical reaction, thereby improving the material’s compactness, electricity, thermal properties, etc. It is an energy-saving and efficient technology of material preparations [8,9]. The preparations of energetic composites by high-energy ball milling method have been reported [10,11]. How to effectively control the morphology and structure of the new energetic composite has not been reported.

In this article, Bi_2_O_3_/Al core-shell energetic composites (hereinafter referred to as Bi_2_O_3_/Al composites) were prepared by means of planetary ball milling in order to introduce Bi_2_O_3_ into the propellant. Based on insensitive HTPE solid propellant (HTPE propellant), the effect of Bi_2_O_3_/Al core-shell energetic composites on the performances of HTPE propellant were investigated as well, which laid a foundation for the application of Bi_2_O_3_/Al core-shell energetic composites in the high energy density solid propellant.

## 2. Materials and Methods

### 2.1. Materials

Aluminum (Al) powder with particle size of 2.7 μm was purchased from Liaoning Angang Industrial Micro Aluminum Powder Co. Ltd. (Anshan, China). Bismuth oxide (Bi_2_O_3_) with a particle size of 6 μm was purchased from Sinopharm Chemical Reagent Co. Ltd. (Shanghai, China). Both Al and Bi_2_O_3_ were dried at 60 °C for 48 h before use. Hexane of analytical purity was used as a coolant in the ball milling process, which was purchased from Beijing Tongguang Fine Chemical Company (Beijing, China).

### 2.2. Two-Step Ball Milling Process

#### 2.2.1. Ball Milling Process

In the first step, a mixture was made by Bi_2_O_3_ powder and hexane in a certain proportion. Then the mixture was put it into the ball milling chamber and ball milled for a certain period of time under certain ball milling conditions. In the second step, Al powder was then added into the ball milling chamber according to the design ratio, and the samples of Bi_2_O_3_/Al composites were obtained after ball milling for a certain period of time. The ball-milled samples were dried in a vacuum oven at 80 °C for 2 h for next testing or using.

#### 2.2.2. Calculation of the Ratio of Al and Bi_2_O_3_

The equation of thermite reaction between Al and Bi_2_O_3_ is as follows:

2Al + Bi_2_O_3_ = Al_2_O_3_ + 2Bi (1)

According to the equation and the molecular weight of Al (26.98) and Bi_2_O_3_ (465.96), the ideal material ratio between Al and Bi_2_O_3_ for ball milling should be:

(2) $$m_{\mathrm{Bi}2O3}=8.64m_{Al}$$

In Equation (2), *m*_Bi2O3_ represents the mass of Bi_2_O_3_ and *m_Al_* represents the mass of Al. In order to facilitate the theoretical calculation, we can assume that the thermite reaction and other chemical reactions do not occur during the ball milling process, but the physical fracture and extrusion deformation of the particles does. The particle size of Bi_2_O_3_ after ball milling in the first step is recorded as *d*, and the particle size of the Bi_2_O_3_/Al composites obtained after ball milling in the second step is recorded as *d_total_*, and the volume of which is recorded as *V_total_*. Thus, we can obtain:

*V_total_* = *V_Al_* + *V*_Bi2O3_ (3)

(4) $$V_{\mathrm{Bi}2O3}=\frac{4}{3}\pi d^{3}$$

From Equations (2)–(4), combining the density of Al and Bi_2_O_3_, we can obtain:

(5) $$d_{total}=1.114d$$

Therefore, the theoretical thickness of the Al coating in the Bi_2_O_3_/Al composite particles is 0.057*d*.

However, in the actual ball milling process, considering the adhesion of a small amount of Al powder on the inner surface of ball milling chamber and the surface of grinding balls [12], not all added Al powder can participate in the preparation of the Bi_2_O_3_/Al composites. According to the surface areas of the inner chamber and the grinding balls, it is assumed that only one layer of Al powder adheres to the inner surface of the chamber and the surface of grinding balls. The utilization rate of the added Al powder can be set to 0.8. Thus, the particle size of the composite particles is:

*d_total_* = 1.139*d* (6)

The actual ratio of Bi_2_O_3_ and Al can be obtained as:

*m*_Bi2O3_ = 6.906*m_Al_* (7)

The theoretical thickness of the Al coating is 0.069*d*.

### 2.3. Instruments and Conditions

A single tank QM-ISP 6planetary ball milling machine, produced by Beijing Feichi Scientific Instrument Co. Ltd. (Beijing, China), was used to fulfill the sample preparation by ball milling. The maximum preparation volume of the ball mill machine is 500 mL.

The morphology characterization and elemental composition analysis of Bi_2_O_3_/Al core-shell energetic composite was performed by an S-4800field emission scanning electron microscope (FESEM), produced by Hitachi Co., Tokyo, Japan. Nano Measure image processing software (Nano Measure 1.2) was used to count the particle size. The number of statistical particles was more than 600.

An X’Pert PRO MPD X-ray diffractometer (Malvern Panalytical, Malvern, UK) was used to analyze the structure and microstructure of energetic composite samples. The test conditions included copper target, KA wavelength of 0.15406 nm, operating voltage of 40 kV, operating current of 40 mA, step size of 0.03°, scanning speed of 2°/min, and test temperature of 25 °C.

The thermal performances analysis of the energetic composite samples were operated by a Swiss METTLER TOLEDO TGA/DSC thermal analyzer (Columbus, OH, USA). Al_2_O_3_ ceramic crucible was used and the sample quality is between 1 and 3 mg. The heating temperature range was 25–800 °C with the heating rate of 10 K·min^−1^. A nitrogen environment was chosen with the nitrogen flow rate of 40 mL·min^−1^.

The density of the samples was analyzed with a densitometer (Dataphysics, Filderstadt, Germany). A Parr 6200 automatic oxygen bomb calorimeter, produced by Anton Paar Co. (Graz, Austria), was used to determine the explosive heat of the HTPE propellant samples containing the Bi_2_O_3_/Al composite.

## 3. Results and Discussion

### 3.1. Optimization of Two-Step Ball Milling Parameters

For the solid particles that undergo thermite reactions, the smaller the particle is, the shorter distances of mass and heat transfer between components will be. In addition, reducing the particle size can increase the surface area of the particles, then lower the ignition temperature and improve the heat release efficiency [13]. Therefore, minimizing particle sizes of the energetic composite particles as much as possible was used as a basis for optimizing ball milling parameters. Since Al is a typical metal material with good ductility, when it is subjected to extrusion and impact, it is easily deformed and combined between particles [14,15]. Bi_2_O_3_ is a typical metal oxide that exhibits significant brittleness and is highly susceptible to particle breakage under the effect of extrusion and impact [16,17]. Therefore, in order to reduce the particle size of the Bi_2_O_3_/Al composite particles, on one hand, the particle size of Bi_2_O_3_ should be minimized firstly. On the other hand, the ball milling parameters should be controlled to obtain uniform Bi_2_O_3_/Al composite particles with minimal sizes. Therefore, a two-step ball milling method was used to prepare the Bi_2_O_3_/Al composites. In the first step, the Bi_2_O_3_ powder was ball-milled with the purpose of reducing the particle size of Bi_2_O_3_. This can avoid the incomplete and uneven refinement of the Bi_2_O_3_ particles due to the energy absorption, which is caused by continuous deformation of Al particles during the one-step ball milling process of the Bi_2_O_3_/Al mixture. Next, in the second step, the blending of Al and ball-milled Bi_2_O_3_ was ball milled again under the optimized conditions. Then, the Bi_2_O_3_/Al core-shell composites were prepared under the effect of the rigidity of the ball-milled Bi_2_O_3_ particles and the extension of Al particles, which can ensure the Bi_2_O_3_ particles are effectively coated by Al.

#### 3.1.1. Ball Milling of Bi_2_O_3_

For the particle samples prepared by the ball milling method, the main factors affecting the particle size are ball milling time, ball milling speed, and ball-to-material ratio. In the first step of the ball milling process, the particle sizes of different Bi_2_O_3_ particles after ball milling under different conditions were investigated. Table 1 shows the statistical results of the particle size of different Bi_2_O_3_ particles at different conditions.

From the results in Table 1, it can be found that the effects of the ball milling parameters on the particle sizes of the samples were different. The degree of the effects of different parameters is as follows: ball milling time > ball-to-material ratio > ball milling speed. The ball milling process mainly produces extrusion, friction, and impact on the samples. Since Bi_2_O_3_ is a brittle material, the pulverization of Bi_2_O_3_ is mainly achieved by impact. Increasing the ball milling time, the ball-to-material ratio, and the ball-milling speed will enhance the effect of the impact. Under the experimental conditions of this study, the ball milling time had the most obvious effect, followed by the ball-to-material ratio and, finally, the ball milling speed. It can also be seen from Table 1 that, under the condition that the ball milling speed and the ball-to-material ratio were constant, the size of the Bi_2_O_3_ particles reaches a minimum of 1.51 μm when the ball milling time was 3 h. Identically, we can determine that the best ball milling parameters include a ball milling speed of 350 r/min, ball-to-material ratio of 15:1, with a ball milling time of 3 h. Under the optimal conditions, the particle size of Bi_2_O_3_ after ball milling is 1.21 μm.

#### 3.1.2. The Preparation of Bi_2_O_3_/Al Core-Shell Energetic Composites

According to the designed material ratio of Al and Bi_2_O_3_, under ideal conditions, Al was finally coated on the surface of Bi_2_O_3_ particles to form a coating with a certain thickness, and the theoretical thickness of this coating was only 0.0685d. That is to say, after the two components were evenly dispersed, the distribution state of the Bi_2_O_3_/Al composites particles was slightly changed compared with that of the single Bi_2_O_3_ particles. Therefore, in the second step of ball milling, we still choose the ball-material ratio and ball milling speed in step one. The particle size statistics of the Bi_2_O_3_/Al composite after ball milling for 1 h, 2 h, and 3 h were counted separately, as shown in Table 2.

**Figure 1:**
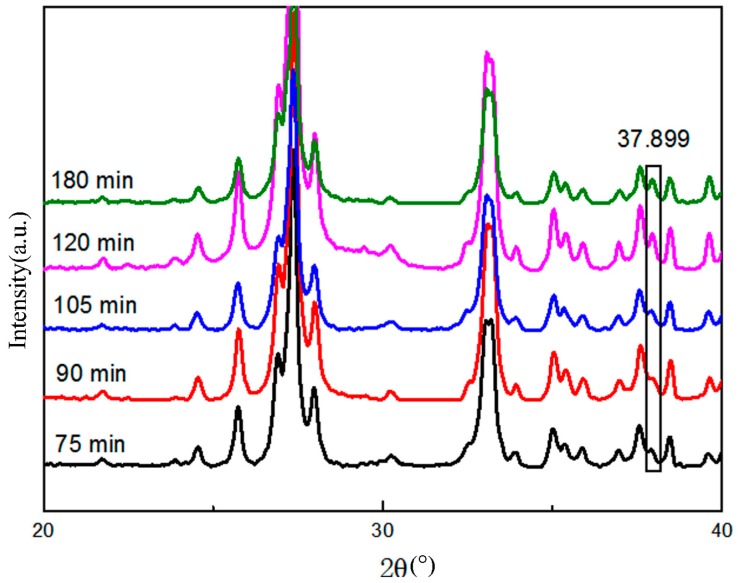
XRD pattern of ball milled samples at different times.

It can be seen from Table 2 that, for the Bi_2_O_3_/Al composite obtained by the two-step ball milling method, when the ball milling time reaches 2 h, the average particle diameter was about 0.40 μm. When the mixing ball milling time was extended to 3 h, the change of particle size was not obvious. We characterized the XRD of the Bi_2_O_3_/Al composite with ball milling time of 75, 90, 105, 120, and 180 min, as shown in Figure 1.

It can be found from Figure 1 that when t ≥ 2 h, the Bi peak appears at 2θ around 37.9°, and the difference of other peaks is not obvious. This indicated that the thermite reaction occurred at some points inside the Bi_2_O_3_/Al composites during the two-step ball milling process when the ball milling time exceeds 2 h. Therefore, when the ball milling time was set to 105 min, the sizes of the particles could be ensured, and the thermite reaction between Al and Bi_2_O_3_ could be avoided.

### 3.2. Morphology and Structure Characterization of Bi_2_O_3_/Al Core-Shell Energetic Composites

#### 3.2.1. Ball Milling Effect and Sample Morphology

**Figure 2:**
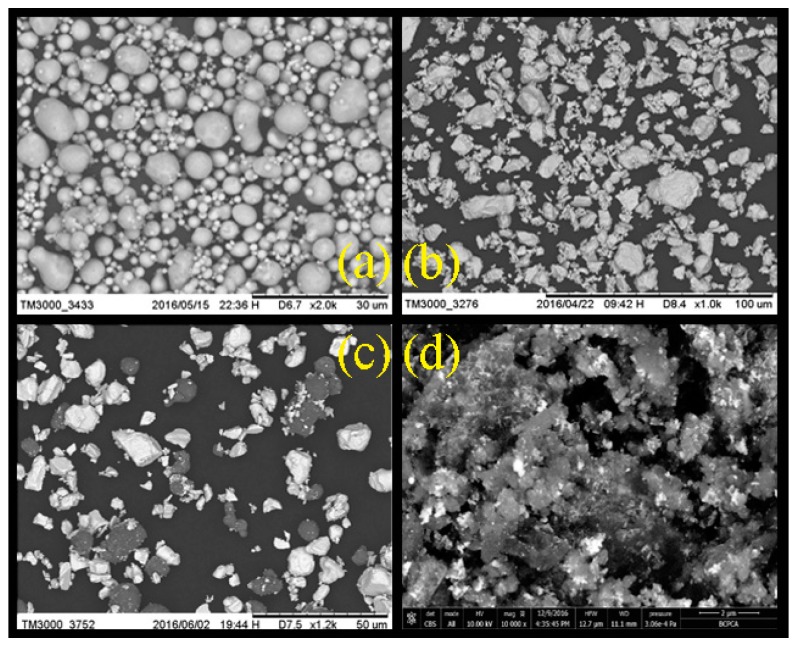
SEM image of sample morphology: (**a**) Al; (**b**) Bi_2_O_3_; (**c**) physical mixing of Bi_2_O_3_ and Al; and (**d**) samples after two-step milling.

Figure 2 showed the SEM images of the raw material of Al and Bi_2_O_3_ without ball milling, the simple physical blending of the two, and the Bi_2_O_3_/Al core-shell energetic composite after two-step ball milling. From which it can be seen that Al particles was spherical, and the shape of Bi_2_O_3_ was irregular. Figure 2c,d shows that, due to the differences in the morphology and atomic number of Al and Bi_2_O_3_, the contrast of the two materials is different, leading to the different brightness of Al and Bi_2_O_3_ in the images. As we can see, Bi_2_O_3_ is brighter than Al. Figure 2c showed that after physical mixing, the morphology of the two had not changed significantly. However, the observed Bi_2_O_3_ in Figure 2d was significantly reduced, indicating that a large amount of Bi_2_O_3_ had entered into the interior of Al to form a core-shell composite. In addition, both crystal forms had undergone significant changes.

**Figure 3:**
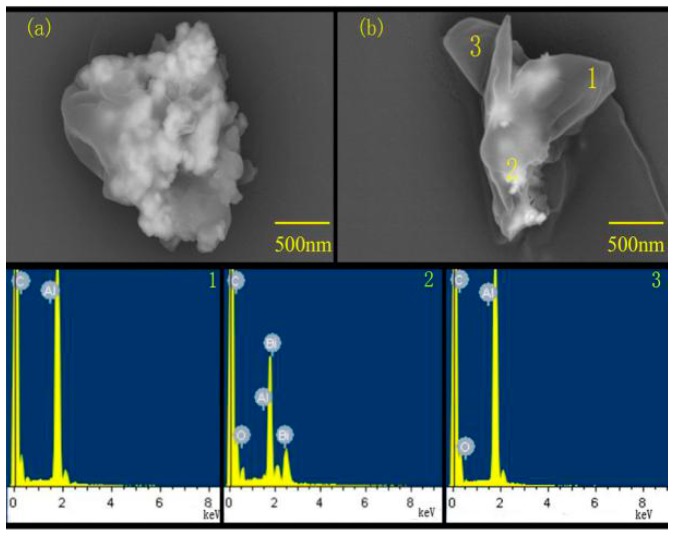
(**a**,**b**) are backscattered scanning electron micrographs; 1, 2, and 3 are, respectively, the EDS spectra of the corresponding positions in Figure 3b.

In order to further determine the structural characteristics and effects of the two-step ball milling method, the EDS spectrum of the points 1, 2, and 3 in Figure 3 were analyzed. It can be seen from Figure 3a,b that the particles were irregular in shape and completely different from the raw materials Al and Bi_2_O_3_. The particles in the figures could be divided into two layers, and the inner layer was brighter and covered with a layer of gray matter. Theoretically, due to the small atomic number of Al and the influence of its composition contrast, the brightness of Al in scanning electron microscopy should be weaker. This inferred that the outer gray material was Al and the inner bright material was Bi_2_O_3_. In Figure 3b, points 1 and 3 have a large gray scale, and the EDS spectrum shows that the elements at the two locations were mainly Al. The brightness at point 2 in Figure 3b is greater, and the EDS spectrum shows that there were a large number of Bi elements in addition to Al. Therefore, the results of the EDS spectrum verify the correctness of the above inference. Combined with the properties of Al and Bi_2_O_3_ and relevant morphology analysis, it can be initially determined that most of the Bi_2_O_3_ particles were entered into the interior of the Al block during the ball milling process, which realizes the final coating of Bi_2_O_3_ with Al, while the thickness of the coating is not uniform. After ball milling, fine Bi_2_O_3_ particles had been extruded into the interior of cold-welded Al particles to form a coated product of Bi_2_O_3_/Al composite particles with core-shell structures. An analysis similar to the related literature can be obtained [18].

#### 3.2.2. Changes in Lattice Structure

**Figure 4:**
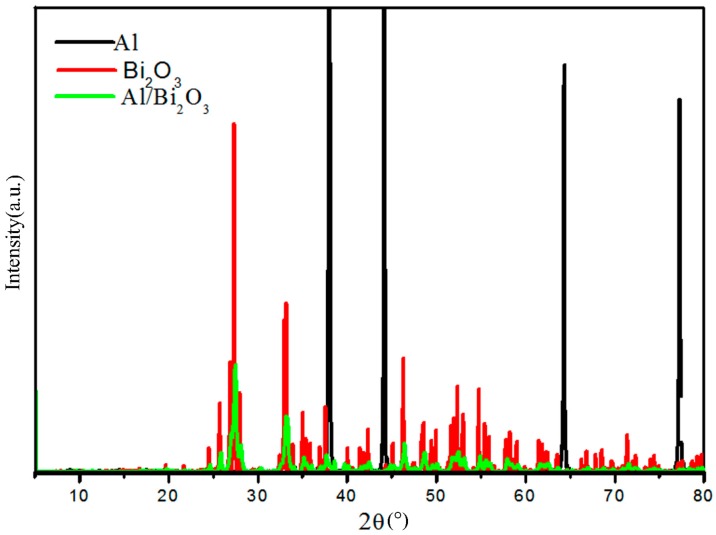
XRD comparison of raw materials and ball-milled samples.

It can be found from the SEM image in Section 3.2.1 that after the two-step ball milling, the morphology of the sample changes greatly, and the lattice structure of the raw material may change greatly or even cease to exist. In order to study the changes in lattice structure, XRD characterization of samples after two-step ball milling was performed and was compared with the XRD of the raw materials, as shown in Figure 4.

XRD can be used to characterize the size of crystals and the degree of crystallization. It can be found in Figure 4 that the characteristic peak of the composite material after ball milling was significantly lower than the characteristic peak of the raw material, indicating that the ball milling had a great influence on the crystal structure of the raw materials. This reason is that during the ball milling processes of the raw materials, the grinding balls had a strong mechanical collision, friction and extrusion on the raw materials, leading to the refinement of the raw material particles and the occurrence of lattice defects, which, in turn, changed the atomic position in the lattice of the materials, and any change in the position of the atom could change the intensity of the diffraction peak [19]. In addition, the decrease in the peak strength of Bi_2_O_3_ was also related to the coating of Bi_2_O_3_ by Al particles.

**Figure 5:**
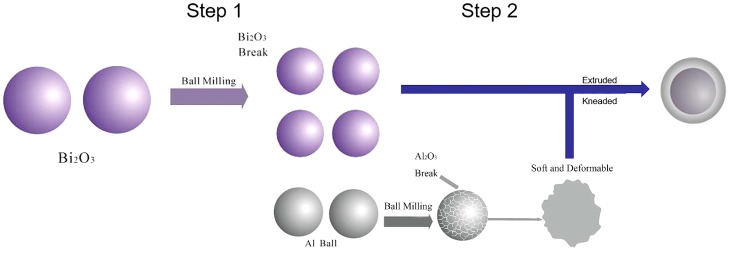
Ball milling process and mechanism diagram.

Combined with the above characterization of the Bi_2_O_3_/Al composite prepared by the two-step ball milling method and the physical properties of the raw materials, we can infer a series of changes that occurred during the ball milling process, which is shown in Figure 5. This description is primarily directed to physical changes during ball milling, assuming that thermite reaction [20,21,22] did not occur. Studies have shown that [23,24,25], under the ball milling conditions, such as longer ball milling time, the thermite reactions could theoretically occur and, indeed, did in practice. In the early stage of ball milling process, the lattice of Al and Bi_2_O_3_ materials was destroyed. Bi_2_O_3_ is a brittle component. Under the action of mechanical force, Bi_2_O_3_ was first broken and dispersed. When Al was added, the hard Al_2_O_3_ shell of Al particles first ruptured and exposed the inner Al after rupture. Since Al itself is a ductile component and the extensibility [26,27] is good, the Al particles undergoes ductile plastic deformation during the ball milling process. Under the action of which, during the process of deformation and calendering, the dispersed Bi_2_O_3_ particles were coated gradually by deformed and extended Al particles, which finally leading to the formation of Bi_2_O_3_/Al core-shell energetic composites. However, since the Bi_2_O_3_ particles have an irregular shape, the coating was not uniform, which can also be found from the SEM images of the samples. We can only theoretically calculate the thickness of the coating under ideal conditions. After ball milling, Bi_2_O_3_/Al core-shell energetic composites had an average diameter of 0.4 μm. Combined with Equation (2.5), we could obtain that the average diameter of Bi_2_O_3_ was 0.351 μm, and the theoretical average thickness of the Al coating was 0.024 μm.

### 3.3. Properties of Solid Propellant Contained Bi_2_O_3_/Al Core-Shell Energetic Composite

#### 3.3.1. Density and Density Specific Impulse of the Propellants

Table 3 shows the density of Bi_2_O_3_/Al energetic composite prepared by two-step ball milling and the physical blending samples of Al and Bi_2_O_3_ in the same mixing ratio.

From Table 3, it can be found that the density of the composite after ball milling is higher than that of the physically blended sample, which is caused by repeated impact, grinding, and stirring during the ball milling process. Due to the continuous compression, some of the particles produced lattice distortion and, at the same time, formed a large number of new active metal surface, then the microscopic particles agglomerated, forming an alloy, resulting in increase of density [28].

Based on the new insensitive HTPE (hydroxyl-terminated polyether) propellant [29], the effects of the application of Bi_2_O_3_/Al core-shell energetic composite on the density and specific impulse of the propellant were calculated and studied. The basic formulation of insensitive HTPE propellant is as follows: the solid content is 80% and the content of Al is 18%, AP (ammonium perchlorate) is 62%, HTPE is 10% and Bu-NENA is 10%. Since the oxidation of Al in the propellant is mainly done by AP, when the Bi_2_O_3_/Al core-shell energetic composite was added to the propellant, the relative content of Bi_2_O_3_ and AP was adjusted accordingly.

It can be seen from Table 4 that for the HTPE propellant systems containing Bi_2_O_3_/Al core-shell energetic composite, with the increase of Bi_2_O_3_ content, the oxygen balance and the theoretical specific impulse decreases linearly, and the density and density specific impulse increase linearly accordingly. Since Bi_2_O_3_/Al is a negative oxygen component, the reduction of AP content inevitably leads to a decrease in oxygen balance. However, since Bi_2_O_3_ is a high-density oxide, an increase in the content of Bi_2_O_3_/Al composite and a decrease in the content of AP led to an increase in the density of the propellant systems. In addition, the theoretical density specific impulse of the propellant systems increases rapidly although the theoretical specific impulse of the propellant systems is reduced with the addition of a large amount of high-density Bi_2_O_3_/Al composite.

#### 3.3.2. Explosion Heat of the Propellant

**Figure 6:**
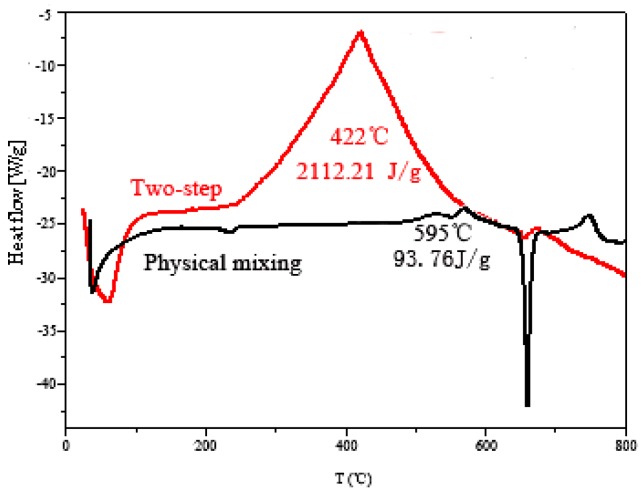
Comparison of DSC curves for different samples.

Firstly, the thermite reactions of the Bi_2_O_3_/Al core-shell energetic composite and the sample of physical mixing at the same material ratio were studied and compared, as shown in Figure 6. It can be seen from Figure 6 that, for the sample of physical mixing, the exothermic peak of the sample appeared at 595 °C, and the calculated exotherm was 93.76 J/g. The exothermic peak of Bi_2_O_3_/Al core-shell energetic composite prepared by two-step ball milling was about 422 °C, and the heat release was as high as 2112.21 J/g, which was very close to the theoretical value of 2118.46 J/g. It is shown that the thermite reaction efficiency of the composite was significantly higher than that of the physically blended sample. This is because, compared with the physically mixed sample, there is a shorter distance of mass and heat transfer and a larger contact area between Bi_2_O_3_ and Al in the composite sample. The core-shell structure makes the contact surface between Bi_2_O_3_ and Al not easy to form a thin layer of Al_2_O_3_, which does not affect the thermite reaction between Bi_2_O_3_ and Al even after long-term storage.

It is worth noting that, comparing the DSC curves of the two samples, the melting peak of Al (660 °C) in the physically mixed sample was sharp and obvious, while the melting peak of Al in the composite was not obvious. This also fully indicated that, in the Bi_2_O_3_/Al core-shell energetic composite, Al and Bi_2_O_3_ underwent a sufficient thermite reaction, and a large amount of Al participated in the reaction and was converted into Al_2_O_3_.

The explosion heat of HTPE propellant systems with different content of ball-milled Bi_2_O_3_/Al core-shell energetic composite were tested and the results were shown in Table 5.

From the data in Table 5, it can be obtained that the explosive heat of the propellant increases with the addition of Bi_2_O_3_/Al core-shell energetic composite. On one hand, the particle size of the Bi_2_O_3_/Al core-shell energetic composite after ball milling is reduced and the mass transfer and heat transfer rate between the components of the system are correspondingly increased, which led to a higher energy release rate. The final performance is that the reaction temperature is lowered and the reaction heat value is increased. On the other hand, the ball milling method itself is a process of energy input, and the energy is released by the explosive reaction of the propellants. Under the combined action of the above factors, when the content of Bi_2_O_3_/Al core-shell energetic composite increases, the explosive heat of the propellants increases.

## 4. Conclusions

In this study, Bi_2_O_3_/Al core-shell energetic composite was prepared by two-step planetary ball milling methods and applied in a HTPE solid propellant. The density of the Bi_2_O_3_/Al composite was increased by about 11.3% compared to the physical mixed sample under the same conditions. The DSC test also showed that the exothermic heat of the thermite reaction of the Bi_2_O_3_/Al composite reached 2112.21 J/g, which was very close to the theoretical exotherm. When the Bi_2_O_3_ content in the system was 19%, the theoretical calculation results showed that replacing the AP with Bi_2_O_3_/Al core-shell energetic composite could make the density of propellant reach 2.056 g/cm^3^ and the density specific impulse reach 502.3 s·g/cm^3^, which was significantly higher than those of the conventional composite solid propellant. The thermal test showed that the explosive heat of the HTPE propellants containing the Bi_2_O_3_/Al composite also increased with increase of the content of the Bi_2_O_3_/Al composite. In addition, we hope to explore the preparation process of spherical or nano-scale Bi_2_O_3_/Al core-shell energetic composite in subsequent research in order to bring better process and mechanical properties to the solid propellants.

## Figures and Tables

**Table 1:** Statistical results of particle sizes of Bi_2_O_3_ particles at different ball milling time.

Ball Milling Time/h	Ball Milling Speed/r·min^−1^	Ball-to-Material Ratio	D_max_/μm	D_min_/μm	D_average_/μm	Standard Deviation
1	350	10:1	7.64	0.45	2.46	0.34
2	350	10:1	6.84	0.37	1.96	
3	350	10:1	3.79	0.26	1.51	
5	350	10:1	4.40	0.32	1.54	
7	350	10:1	5.06	0.32	1.56	
9	350	10:1	6.24	0.33	1.94	
3	250	10:1	6.66	0.33	1.67	0.06
3	300	10:1	6.31	0.28	1.55	
3	350	10:1	3.79	0.26	1.51	
3	400	10:1	5.04	0.20	1.52	
3	450	10:1	6.38	0.22	1.63	
3	350	5:1	6.66	0.30	1.64	0.14
3	350	10:1	3.79	0.26	1.51	
3	350	15:1	3.28	0.22	1.21	
3	350	20:1	3.65	0.22	1.39	
3	350	25:1	4.38	0.23	1.45	

**Table 2:** Statistical results of particle size.

Ball Milling Time/h	D_max_/μm	D_min_/μm	D_average_/μm
1	4.83	0.34	0.99
2	3.52	0.13	0.40
3	3.43	0.10	0.35

**Table 3:** Density of different simples.

Sample	Density/g·cm^−3^
Al	2.5587 ± 0.0132
Bi_2_O_3_	8.7963 ± 0.0161
Physical mixing	7.1880 ± 0.0147
Step ball mill	8.0030 ± 0.0129

**Table 4:** Effect of Bi_2_O_3_ content on energy performances of the HTPE propellant systems with the same solid content.

Bi_2_O (%)	AP (%)	OB	ρ (g/cm^3^)	I_SP_ (s)	I_SR_ (g·s/cm^3^)
0	62	0.596	1.778	269.2	478.6
2	60	0.585	1.804	266.7	481.1
4	58	0.575	1.830	264.2	483.5
6	56	0.564	1.857	261.7	486.0
8	54	0.553	1.885	259.1	488.5
10	52	0.542	1.914	256.5	491.0
12	50	0.531	1.944	253.9	493.5
15	47	0.514	1.991	249.8	497.3
17	45	0.503	2.023	247.1	499.8
19	43	0.491	2.056	244.3	502.3
20	42	0.485	2.073	242.9	503.5
21	41	0.480	2.091	241.4	504.7
23	39	0.468	2.126	238.5	507.0
25	37	0.456	2.163	235.2	508.8
26	36	0.450	2.182	233.4	509.3

*I_SP_* is the theoretical specific impulse. *I_SR_* is the theoretical density specific impulse.

**Table 5:** Explosion heat of HTPE propellants containing different contents of Bi_2_O_3_/Al composite.

Content of Bi_2_O_3_/Al Composite in HTPE Propellant/wt%	Explosive Heat/MJ·kg^−1^
0	4.29
5	4.39
7.5	4.45
10	4.91
18	5.03
21	5.20
